# The effect of improved dietary control on cognitive and psychiatric functioning in adults with phenylketonuria: the ReDAPT study

**DOI:** 10.1186/s13023-020-01668-2

**Published:** 2021-01-18

**Authors:** Nicholas M. Burgess, Wendy Kelso, Charles B. Malpas, Toby Winton-Brown, Timothy Fazio, Julie Panetta, Gerard De Jong, Joanna Neath, Sonny Atherton, Dennis Velakoulis, Mark Walterfang

**Affiliations:** 1 Neuropsychiatry Unit, Royal Melbourne Hospital, Level 2, John Cade Building, Melbourne,, 3050 Australia; 2 Department of Neurology, Royal Melbourne Hospital, Melbourne, Australia; 3 Clinical Outcomes Research Unit (CORe), Department of Medicine, Royal Melbourne Hospital, The University of Melbourne, Melbourne, Australia; 4 Department of Neuroscience, Central Clinical School, Monash University, Melbourne, Australia; 5 Department of Metabolic Medicine, Royal Melbourne Hospital, Melbourne, Australia; 6 Melbourne Medical School, Department of Medicine and Radiology, University of Melbourne, Parkville, Australia; 7 Gold Coast University Hospital, Southport, Australia; 8 Melbourne Neuropsychiatry Centre, University of Melbourne and North-Western Mental Health, Melbourne, Australia; 9 Florey Institute of Neuroscience and Mental Health, Melbourne, Australia

**Keywords:** Phenylketonuria, Diet, Cognitive function, Processing speed, Depression, Anxiety

## Abstract

**Background:**

Phenylketonuria (PKU) is an autosomal recessive inherited disorder characterised by a deficiency in phenylalanine hydroxylase. Untreated, PKU is associated with a wide range of cognitive and psychiatric sequelae. Contemporary management guidelines recommend lifetime dietary control of phenylalanine (Phe) levels, however many individuals who discontinue dietary control subsequently suffer symptoms of anxiety, depression and disturbances to cognition. We undertook a prospective cohort study of patients with early-treated phenylketonuria who had ceased dietary control to test the hypothesis that resumption of dietary control of PKU is associated with improvements in measures of psychiatric morbidity and cognitive functioning.

**Methods:**

We re-initiated dietary control for early-treated patients with PKU and monitored cognitive and psychiatric outcomes over a twelve-month period. Assessments included objective cognitive function (measured by cognitive proficiency index (CPI)), anxiety and depression scales. General linear mixed model (GLMM) analyses were performed to assess change in psychometric variables from baseline over twelve months after resumption of dietary control.

**Results:**

A total of nine patients were recruited. Mean age was 33 years (SD = 8.75), five were female. Mean time off dietary control was 19.1 years (SD = 11.3), and mean baseline phenylalanine (Phe) levels were 1108 µmol/L (SD = 293). GLMM analysis demonstrated a positive relationship between CPI and time on diet (*b* = 0.56 [95% CI = 0.17, 0.95]). Age, time off diet, Phe levels and depression scores were not associated with cognitive function. There was a negative relationship between time on diet and anxiety (*b* = − 0.88 95% CI = [− 1.26, − 0.50]) and depression ratings (*b* = − 0.61, 95% CI = [− 0.95, − 0.26]).

**Conclusions:**

This study demonstrated improvements in cognitive function, anxiety, and depression ratings associated with resumption of dietary control of PKU. Raw Phe levels were not strongly associated with psychiatric or cognitive scores in this cohort. These findings support the importance of lifelong treatment for PKU in improving the cognitive and psychiatric sequelae of the disease.

## Introduction

Phenylketonuria (PKU) is an autosomal recessive inherited disorder characterised by a deficiency in phenylalanine hydroxylase (PAH) associated with a wide range of cognitive and psychiatric sequelae [1]. PKU is the most commonly occurring inborn error of amino acid metabolism and is the result of one of more than 950 pathological variants in the PAH gene [2]. PAH is responsible for converting phenylalanine (Phe) to tyrosine. Phe levels are elevated in patients with PKU due to deficiencies in PAH activity, resulting in toxic accumulation of Phe in brain tissues.

Hyperphenylalaninaemic (hyperPhe) states have been found to cause disruptions to neurotransmission.

At high concentrations, Phe competes with other large neutral amino acids (LNAA) -tyrosine, tryptophan, valine, isoleucine, leucine, threonine, methionine, and histidine—at the L-type amino acid transporter (LAT1) for transport to the brain across the blood–brain barrier (BBB). In PKU, disruption of relative concentrations of amino acids (hyperPhe and depletion of other LNAAs) results in saturation of the transporter by Phe, and reduced transport of other LNAAs [3]. Heightened concentrations of Phe have been implicated in directly decreasing cerebral protein synthesis, leading to overall disruptions in neurotransmitter activity [4].

Tyrosine and tryptophan are precursor molecules for the synthesis of key neurotransmitters such as serotonin, dopamine and norepinephrine, which play key roles in cognition. Dopamine is strongly implicated in the control of executive functioning in the prefrontal cortex through modulating responses to salient stimuli, allowing for effective allocation of attention, working memory and planning [5]. Serotonin has both pro-cognitive and neuroprotective effects, and its activity is widely distributed throughout the CNS. It has important roles in neuro-modulatory control in prefrontal regions as well as in mood and anxiety regulation in limbic structures [6, 7]. As such, disruption to monoamine levels as a result of untreated PKU has significant implications as a cause of neuropsychiatric disability in patients with the disease [8–10].

Dietary control has long been the mainstay of treatment for PKU. The current European guidelines [11] on PKU define dietary control as consisting of three components: (I) the restriction of natural protein, (II) use of Phe-free-L-amino acid supplements, and (III) prescription of specially formulated low-protein foods. By restricting proteinaceous foods high in Phe such as meat, poultry, fish, eggs, legumes and cereals [11], the diet aims to maintain low Phe levels whilst minimising risk of nitrogen deficiency and maintaining body mass. The diet is supplemented by with Phe-free-L-amino acids to minimise the risk of deficiency. The ‘PKU diet’ has formed the cornerstone of treatment and was traditionally maintained only during critical periods of neurodevelopment. In the United States, traditional treatment paradigms supported dietary control being maintained to six years of age, though over the years this juncture was extended to twelve years as benefits to neurodevelopment were demonstrated [12]. More recent European guidelines have recommended lifelong adherence to dietary control in light of evidence of associations with improved neuropsychiatric symptoms and continuous control [11].

PKU can result in a broad constellation of cognitive and psychiatric symptoms. A 2007 meta-analysis demonstrated a significant relationship between poor Phe control and general intellectual abilities, with each 100 µmol/L predicting a 1.3 to 3.9 reduction in the intelligence quotient (IQ) for early treated patients with PKU [13]. Adults with PKU also have significantly impaired processing speed as compared to healthy controls, and in complex executive functioning – particularly in tasks involving planning and cognitive flexibility [14, 15], and attract higher rates of diagnoses of intellectual disability and autism [16]. The sequelae of poor dietary control in early-treated adults appear to be at least partially reversible. Improvements in white matter/structural lesions [17], and cognitive sequelae [18, 19] have all been demonstrated with Phe control and dietary supplementation. Depressive/anxiety symptoms have been correlated with Phe levels [20]. Quality of life measures improve with the resumption of dietary control [18, 21], altogether adding weight to the importance of lifetime control [11].

Many adult patients initiated on dietary treatment from infancy (early-treated), who subsequently discontinued or relaxed their dietary control experience a significant burden of cognitive, neurological, psychiatric symptoms as well as worsening psychological wellbeing [12, 22–24]. In this observational study the “Resumption of Diet control in Adult Phenylketonuria Trial” (ReDAPT), we assess the effects of resumption of the PKU diet in previously early-treated adults on both cognitive and psychiatric profiles.

## Methods

### Participants

The sample included nine subjects recruited from the Metabolic Service at the Royal Melbourne Hospital, with confirmed PKU (by neonatal Guthrie card or genetic testing) who had been initiated on dietary treatment during childhood and ceased prior to 18 years of age and off diet for at least 5 years. Subjects were excluded on the basis of MRI contraindication, intellectual disability, or comorbid drug or alcohol dependence. If meeting these criteria, patients were referred to the neuropsychiatry unit for consent and recruitment into the study. Research and ethics approval for the project was provided by the Melbourne Health Human Research Ethics Committee (MH Project Number 2014.113).

### Metabolic Assessment

Each patient underwent baseline metabolic assessment, with initial review by a metabolic physician and dietician for review of PKU history Serology for baseline plasma Phe (Australian reference range 120–360 µmol/L) and Tyr levels (reference range 40–80 µmol/L) were taken by blood test and analysed by Victorian Clinical Genetics Services, with repeat measures at 6 and 12-month timepoints. Patients were guided by metabolic dieticians on dietary management of PKU, with re-initiation of dietary control and supplementation as necessary. Supplementation included combinations of glycomacropeptide and essential amino acids with vitamins and minerals, adjusted to suit the requirements of each individual based on patient reported intake and most recent Phe & Tyr measurements. Dietary adherence was assessed by patient reports of typical diet, and serology results. Phe levels and diet were reviewed as part of routine clinical care in metabolic diseases outpatient clinics.

### Psychiatric assessment

Each patient underwent a baseline psychiatric assessment by a neuropsychiatrist who completed and scored the Hamilton Anxiety Rating Scales (HAM-A), (with scores of 8–14 = mild anxiety, 15–23 = moderate anxiety, ≥ 24 = severe anxiety) and the Hamilton Depression Rating Scales (HAM-D), with scores of 8–13 = mild depressive symptoms, 14–18 = moderate, 19–22 = severe and ≥ 23 = very severe. Repeat assessments were completed at 6- and 12-months post resumption of diet.

### Neuropsychological assessment

Each participant underwent baseline neuropsychological assessment by a clinical neuropsychologist. Attention/working memory as well as processing speed were assessed with completion of the Weschler Adult Intelligence Scale-IV (WAIS-IV) digit span, arithmetic, coding and symbol search subtests. The cognitive proficiency index (CPI) is a derivation of WAIS subtests that focuses on the proficiency and efficiency of cognitive processing [25], that has been shown to be exquisitely sensitive to the disruption of diffuse neurocognitive networks [26]. It provides a normed inded score of 100 (± SD = 15). Repeat assessments were completed at 6- and 12-months post resumption of diet. At baseline only, each participant completed the Wechsler Test of Adult Reading (WTAR) to estimate pre-morbid IQ, with a calculated normed index score of 100 (± SD = 15).

### Statistical analysis

All statistical analyses were performed using the *jamovi* software package which runs on *R* [27]. Descriptive statistics are presented as counts, means, and standard deviations where appropriate. Longitudinal change in psychometric markers was investigated using general linear mixed-effects models (GLMMs) implemented using the GAMLj package [28]. For each marker, the psychometric score was entered as the dependent variable. Months on diet post adult resumption of dietary control was entered as the primary independent variable of interest. Gender, number of years off diet, and Phe levels were entered as adjustment variables. The HAM-D score was added as an adjustment variable for the CPI analysis. Continuous variables were centered prior to analysis. A random intercept was specified for each participant. Random slopes for time were considered, but these models did not converge consistently. As such, the more parsimonious fixed slope models are reported. All models were estimated using restricted maximum likelihood. Parameter estimates with 95% confidence intervals (CI) are reported for statistical inference.

## Results

### Baseline sample characteristics

The clinical characteristics of the patients included in the study are shown in Table 1. The sample was comprised of data from 9 patients, 5 of whom were female. Ages ranged from 19 to 47, with a mean of 34 year of age. The mean period of time since dietary control had been ceased was 19.1 years (*SD* = 11.3). Mean Phe levels at baseline were 1108 µmol/L (*SD* = 293 µmol/L). IQ as estimated by the WTAR at baseline was 96.9 (*SD* = 5.20). Baseline scores for anxiety as rated on the HAM-A ranged from 5 (normal to mild range) to 28 (within the ‘severe’ range), mean score was 18.6 (*SD* = 8.03). Scores for depression at commencement of the trial were generally in the mild-moderate range, with a mean of 13.1 (*SD* = 5.82). One patient withdrew from the study after the initial assessment and was lost to follow-up. All other patients remained engaged in the study, and reported dietary adherence. Two patients were on antidepressant medications, and one of these also was taking a mood stabiliser, these patients did not demonstrate trends that differed to other participants. The baseline CPI scores (derived from WAIS-IV scores) had a mean value of 91.9 (*SD* = 8.21).

**Table 1:** Sample characteristics, group and individual, Mean (*SD*) unless specified

Participant	Female (%)	Age (y)	Years off diet	Phe µmol/L	HAM-A	HAM-D	WTAR	CPI
Overall	5 (55.6)	33 (8.75)	19.1 (11.3)	1108 (293)	18.6 (8.03)	13.1 (5.82)	96.9 (5.20)	91.9 (8.21)
1	Female	33	26	814	28	22	106	80
2	Female	35	25	1038	23	17	103	96
3	Male	35	30	728	-	-	109	102
4	Female	42	10	1121	14	15	92	80
5	Female	24	5	1602	25	14	90	96
6	Male	19	5	931	22	16	96	99
7	Male	27	10	1186	5	4	60	89
8	Male	47	30	1043	22	10	108	98
9	Female	38	31	1513	10	7	108	87

Phe, Phenylalanine level; HAM-A, Hamilton Anxiety Rating Scale; HAM-D, Hamilton Depression Rating Scale; WTAR, Weschler Test of Adult Reading; CPI, Cognitive Proficiency Index

### Cognitive function

**Fig. 1:**
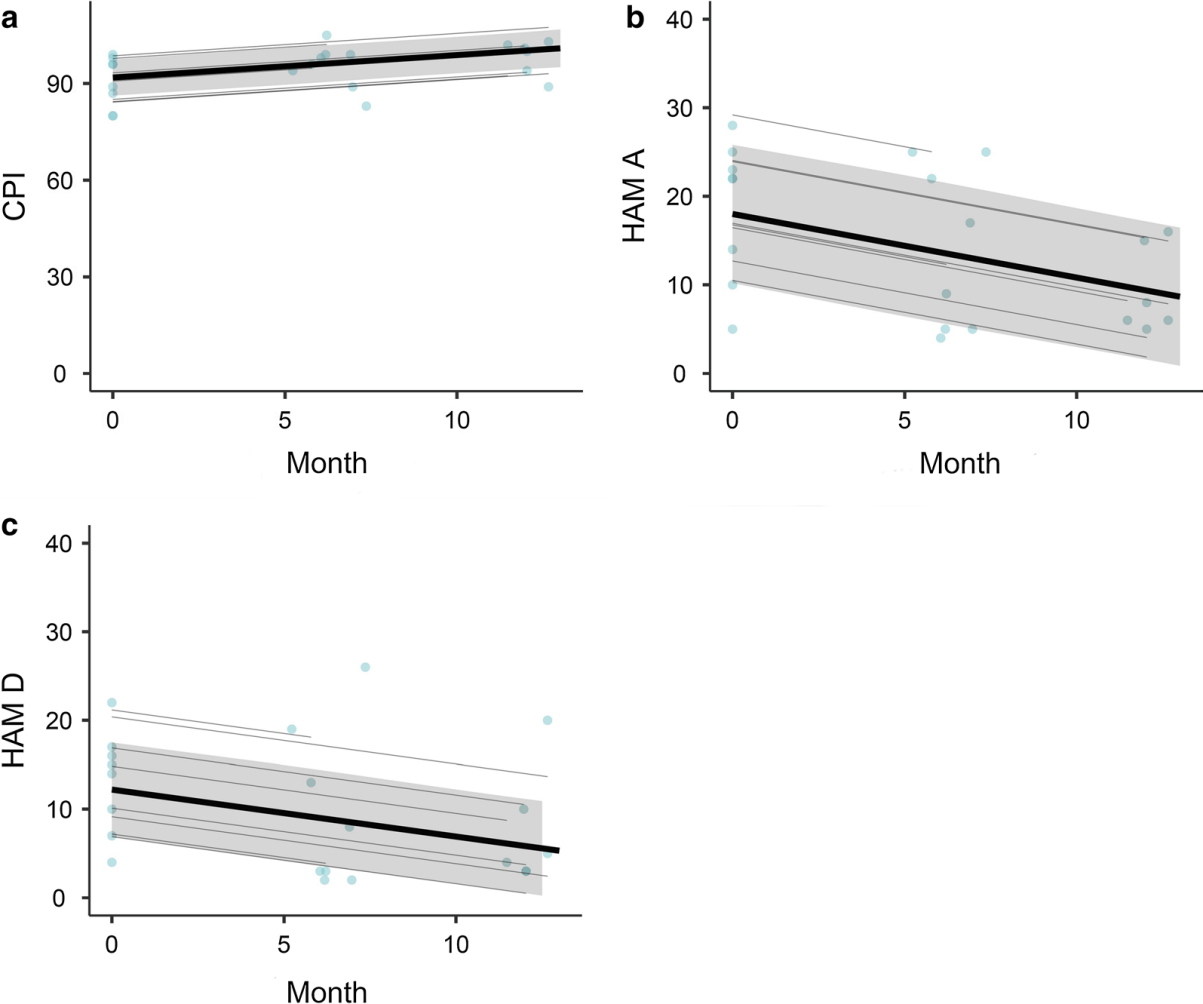
**a** Effects plots for time (months) on diet vs CPI score (95% confidence interval shaded). **b** Effects plots for time (months) on diet vs HAM-A score (95% confidence interval shaded). **c** Effects plots for time (months) on diet vs HAM-D score (95% confidence interval shaded). HAM-A, Hamilton Anxiety Rating Scale; HAM-D, Hamilton Depression Rating Scale; CPI, Cognitive Proficiency Index

Cognitive function (as measured by CPI) was assessed at baseline, 6 and twelve months as part of neuropsychological assessment (Table 2). Mean CPI score improved from 91.9 (SD = 8.21) to 99.4 (SD = 5.40) across the twelve-month trial period. As shown in Table 3, the GLMM revealed that time on diet (months) was positively associated with cognitive function, *b* = 0.70 [95% CI = 0.35, 1.04]. Gender, years off diet, Phe levels, and HAM-D scores were not significant predictors. We repeated these analyses for the PSI and WMI indices separately, which produced comparable results. The effect of time on diet persisted when all other variables (gender, years off diet, Phe levels, and HAM-D scores were not significant predictors) were excluded from the model, *b* = 0.71 [95% CI = 0.48, 0.93]. Figure 1 (A) shows the change in CPI over time. Scores for CPI as compared to population norms increased from the 27th percentile on average to the 47th percentile on average at twelve months.

**Table 2:** Clinical variables against time on PKU diet—M (*SD*)

Clinical variable	Baseline	6 Months	12 Months
HAM-A	18.6 (8.03)	14.0 (9.27)	9.33 (4.89)
HAM-D	13.1 (5.82)	9.50 (9.06)	7.50 (6.66)
CPI	91.9 (8.21)	95.4 (6.78)	99.4 (5.40)
PSI	94.9 (8.65)	97.3 (7.89)	106 (8.09)
WMI	90.7 (8.69)	94.1 (9.51)	93.6 (6.48)
Delta-Phe	0 (0)	− 476 (290)	− 372 (250)

HAM-A, Hamilton Anxiety Rating Scale; HAM-D, Hamilton Depression Rating Scale; CPI, Cognitive Proficiency Index; PSI, Processing Speed Index; WMI, Working Memory Index; Delta-Phe, Change in Phenylalanine

**Table 3:** Model parameters – *b* [95% CIs]

Term	CPI	HAM-A	HAM-D
*Adjusted*			
Gender (male)	8.64 [− 0.65, 17.92]	− 6.06 [− 18.40, 5.35]	− 8.93 [− 17.03, − 0.84]
Years off diet	0.19 [− 0.24, 0.63]	− 0.04 [− 0.65, 0.57]	0.21 [− 0.47, 0.36]
Phe	0.01 [− 0.00, 0.03]	− 0.01 [− 0.04, 0.01]	− 0.02 [− 0.03, 0.00]
HAM-D	− 0.13 [− 0.57, 0.30]	–	–
Time on diet	0.70 [0.35, 1.04]	− 0.72 [− 1.07, − 0.37]	− 0.53 [− 0.82, − 0.24]
*Unadjusted*			
Time on diet	0.71 [0.48, 0.93]	− 0.71 [− 1.06, − 0.36]	− 0.51 [− 0.81, − 0.22]

Phe, Phenylalanine level; HAM-A, Hamilton Anxiety Rating Scale; HAM-D, Hamilton Depression Rating Scale; CPI, Cognitive Proficiency Index. Estimates are model parameters [95% CIs]

### Psychiatric measures

As show in Table 3, time on diet was negative associated with HAM-A scores, *b* = − 0.72 [95% CI = − 1.07, − 0.37]. The effects of gender, Phe levels, and years off diet were not significant predictors. The effect of time on diet persisted when all other variables (gender, years off diet, Phe levels) were excluded from the model, *b* = − 0.71 [95% CI = − 1.06, -0.36]. A similar pattern was observed for HAM-D levels. While time on diet was negatively associated with HAM-D scores, *b* = -0.53 [95% CI = − 0.82, − 0.24], there was no evidence for the effect of gender, Phe levels, and years off diet. This effect remained unchanged for time on diet when all other variables (gender, years off diet, Phe levels) were excluded from the model, *b* = − 0.51 [95% CI = − 0.81, − 0.22]. These relationships are shown in Fig. 1b and b. Within our cohort, one patient did not complete depression or anxiety screening tools. For ratings of anxiety on the HAM-A, those who screened positive for severe anxiety at twelve months had scores falling within the moderate range. Of those who scored within the moderate range, one had reductions from the high end of moderate [23] to the low end [15], one scored within the mild range, and one the normal range. Of those whose screening demonstrated mild anxiety at baseline, at twelve months scored within the normal range. One participant who did not screen positive for anxiety at twelve months scored at the low end of the cutoff for mild anxiety [8]. No patients screened within the severe range for depression on the HAM-D at baseline. Of those who scored within the moderate range, one did not report a significant change in symptoms at twelve months, one scored within the mild range, and one in the normal range. For those whose screening at baseline placed them within the mild range of depressive symptoms, one remained within the mild range at twelve months, and two within the normal range. Of patients who did not screened within the normal range for depressive symptoms at baseline, all remained in the normal range at twelve months.

## Discussion

PKU causes significant cognitive and psychiatric morbidity and worsening quality of life through a number of mediating factors. Though current guidelines reflect the established move towards lifelong dietary control rather than cessation after critical periods of neurodevelopment, individual patients may have received differing advice on when to cease or continue a Phe-restricted diet depending on their age and locale. As a result, many adults with PKU have ceased the PKU diet either through medical advice or personal preference. In general, motivation of patients to maintain adherence to the PKU diet is often suboptimal, and likely worsened by deficits in executive function and poor mood as direct sequelae of the disease [29]. In this study, we evaluated the cognitive and psychiatric effects of resumption of dietary control of phenylketonuria in adult patients who had previously ceased dietary control to determine if these symptoms could be improved.

Across the twelve-month study duration, we found that resumption of the PKU diet was associated with improvements in symptoms of depression, anxiety, and measures of cognitive functioning. Multiple mechanisms may underlie such improvements. Firstly, the restoration of central levels of key monoamines, results in a relative normalisation of monoaminergic tone and thus a reduction in dimensional levels of depression and anxiety. Secondly, the attenuation of impaired myelination (itself intimately related to monoaminergic signaling), improves cognitive functions that are underpinned by more widely distributed cortico-cortical and cortico-subcortical networks, such as working memory and processing speed [8]. Improvements to cognitive functioning were not explained by improvement in mood, with only time on diet being a significant covariate. Processing speed (in this study captured by CPI scores), which is well recognised as being reduced in adults with PKU [14, 30], is centrally mediated and likely reflective of the underlying dysmyelination that has been observed in PKU [31]. Assessing neurological structural changes in this cohort may further shed light on the underlying mechanisms of our findings.

Whilst the association between PKU and psychiatric and cognitive disturbance has been well established, the total time on diet following resumption, more so than serum Phe, appeared to be the most significant factor in determining improvements in cognition or mood and anxiety symptoms in our cohort. This apparent lack of correlation may also be explained by the cross-sectional nature of the Phe measurements. Phe may fluctuate significantly day-to-day in patients with even well controlled PKU [32], and it may be that the time-on-diet metric was acting as a proxy measure of Phe levels as an average over time. The lack of correlation between cognitive or mood symptoms with Phe has been observed previously [33], and may be more reflective of improvements to white matter integrity with dietary control rather than contemporaneous Phe levels. This would be in keeping with previous studies which have demonstrated improvements to hypomyelination in individuals who have resumed dietary control [34]. Our findings are consistent with previous studies suggesting at least partial reversibility of cognitive and psychiatric deficits in adults with PKU when dietary control is resumed or improved, and are broadly consistent with the notion that clinical goals in PKU should include maintaining Phe within normal ranges lifelong as this will result in significantly improved cognitive and psychiatric outcomes.

Though the benefits to psychiatric symptoms and cognition are likely to improve the overall wellbeing of patients, the task of maintaining adherence to the PKU diet can be onerous and frustrating for those who miss the restricted foods, or find supplementation products unpleasant or distasteful [35]. High rates of attritional loss to follow-up of patients in metabolic clinics has also been recognized [36, 37], likely in part owing to the neuropsychiatric sequelae of the disease and further heightens the risk of ongoing morbidity from the disease. Further advances in pharmacological management of PKU offer hope to those who find dietary control unachievable whilst allowing for improved quality of life. Tetrahydrobiopterin (BH4), an essential co-factor for PAH in the metabolism of Phe to Tyr [38] has been utilised as a treatment option for PKU as sapropterin dihydrochloride. Administration of sapropterin has been found to be effective in reducing serum Phe by ≥ 30% from baseline in up to half of those treated [39], though patients with milder PKU were more likely to derive benefit [40]. Novel emergent therapies such as the recently FDA approved PEGylated adducts of phenylalanine ammonia lyase have recently been shown to be efficacious in reducing Phe levels [41, 42], and may offer alternatives or adjuncts to dietary control as the mainstay of management.

The rigours of maintaining the PKU diet may in themselves prove detrimental to a sense of wellbeing, and despite the well documented benefits to neuropsychiatric symptoms, may not improve overall subjective quality of life. In a study of quality of life in those restarting the PKU diet, though 60% found that adherence conferred overall benefits, 40% felt that their overall quality of life was unchanged despite restarting dietary control [35].

Our study was limited by a relatively small sample size and would benefit from replication on a larger scale. The longitudinal nature of the study, however, mitigates against this risk. The use of confidence intervals allowed us to understand the effect that the small sample size had on parameter uncertainty, which was relatively minimal. Whilst significant improvements in anxiety and depression were observed in the group, the majority of participants endorsed mild or moderate symptoms—inclusion in future studies of those with more severe psychopathology may potentially yield greater results. Of particular note, the low-frequency testing of Phe levels may result in measurements not representative of average Phe as individual variance in day-to-day Phe levels can be large. This may in part explain the lack of a clear association between Phe levels and the significant improvement in measures of anxiety and depression symptoms and cognitive outcomes. Our study also excluded individuals with an intellectual disability, which may bias sampling and impact outcomes particularly regarding cognitive measures. During the study one patient had their antidepressant changed by their primary care physician, and a second commenced an SSRI which may have had a small effect on overall results. Other, less readily measurable benefits were not necessarily captured in our study design. Within our own cohort, individual feedback in some cases was of subtle improvements to domains such as improved frustration tolerance, improved concentration and improvements in skin complexion. These benefits may not necessarily be captured in what can be blunt measurement tools.

## Conclusion

Our findings demonstrate that early-treated patients who resumed dietary control of PKU had improvements in both cognitive and psychiatric measures. Specifically, improvements were observed in cognitive functioning as derived by CPI, and in the participants’ clinical scores of depression and anxiety on HAM-D and HAM-A rating scales. These data highlight the importance of regular follow up and screening for what can often be subtle psychiatric or cognitive disorders in individuals with PKU, especially in those who have ceased or relaxed dietary control. Ideally, dietary control should be continued for life, however personal preferences and circumstances also need to be considered. Individuals who have ceased the PKU diet previously should be counselled on the reversibility of many of the sequelae of PKU as part of routine care.

## Data Availability

The datasets used and/or analysed during the current study are available from the corresponding author on reasonable request.
